# Editorial: Intracoronary Imaging Guidance for Percutaneous Coronary Interventions

**DOI:** 10.3389/fcvm.2022.944853

**Published:** 2022-05-27

**Authors:** Gianluca Caiazzo, Alessio Mattesini, Paolo Canova, Ismail D. Kilic

**Affiliations:** ^1^San Giuseppe Moscati Hospital, Aversa, Italy; ^2^Careggi University Hospital, Florence, Italy; ^3^Papa Giovanni XXIII Hospital, Bergamo, Italy; ^4^Pamukkale University, Denizli, Turkey

**Keywords:** intracoronary imaging, PCI, OCT, IVUS, NIRS

Technology revolutionizes the way we practice medicine. This is also true for percutaneous coronary interventions (PCI). From intravascular ultrasound (IVUS) and optical coherence tomography (OCT) to spectroscopy or florescence techniques, intracoronary imaging (ICI) represents one of the ultimate examples of the combination of Cardiology and technology. These modalities describe the various aspects of the healthy or diseased arterial wall and allows to perform coronary interventions in great detail, unlike conventional angiography (Figure 1). Aiming at achieving optimal results in a constantly increasing percentage of cases should be the target of Interventional Cardiologists, especially in those anatomical settings where coronary artery by-pass graft surgery (CABG) still represents the gold standard therapeutic option. In the last two decades, it has been widely demonstrated that the use of intracoronary imaging (ICI) positively and significantly impacts the clinical outcomes of the patients undergoing PCI procedures. In the “*Intracoronary Imaging Guidance for Percutaneous Coronary Interventions*” Research Topic in Frontiers in Cardiovascular Medicine, several studies underlining the value of ICI in the context of PCIs and offering a wide spectrum of detailed imaging-based analyses of both atherosclerotic and non-atherosclerotic coronary lesions were collected. The main goal is to provide useful insights in order to help the readers understand the aspects of intracoronary imaging techniques that positively impact PCIs the most, as well as the procedural phases of coronary angioplasty where these tools are helpful. Moreover, this collection provides a spotlight on imaging-related technological novelties and analytic systems, as well as new evidences on the clinical impact of intracoronary imaging use during PCIs.

**Figure 1:**
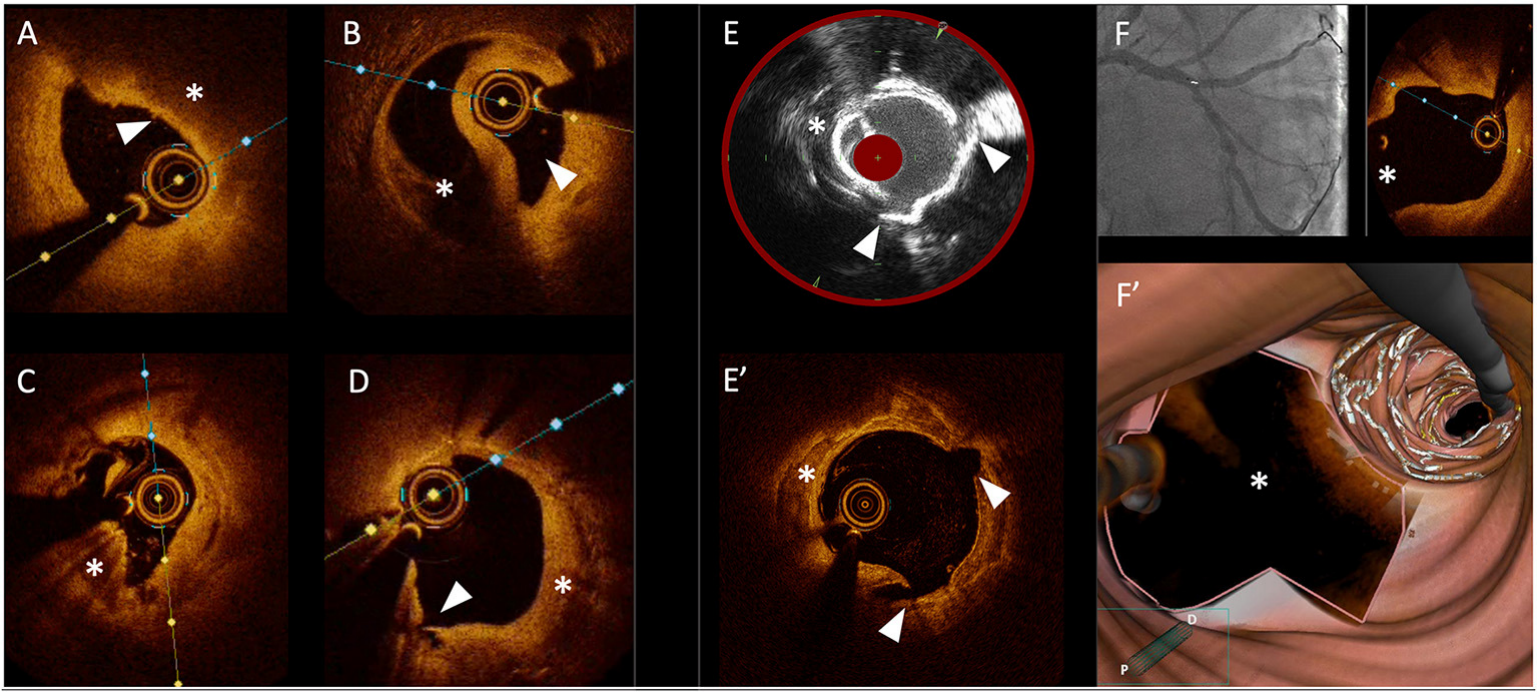
Gallery of images obtained with different intracoronary imaging tools. **(A)** OCT cross section showing a coronary plaque at high-risk of rupture with a thin-cap (arrow head) covering a lipid-rich atheroma. **(B)** OCT cross section showing a coronary dissection with a clear distinction of the true lumen (arrow head) and the false lumen (asterisk). **(C)** OCT cross section showing a calcified nodule (asterisk). **(D)** OCT cross section showing in-stent neointimal growth (asterisk) causing re-stenosis; the arrow head shows a small intimal dissection. **(E)** Optimal correspondence between images obtained with IVUS/NIRS **(E)** and OCT **(E')** showing a cracked calcific plaque after treatment with intravascular lithotripsy (with arrow heads showing corresponding cleavage planes and asterisks showing a calcific plaque). **(F)** OCT pullback showing a precise ostial stent implantation with no stent struts protruding into the main vessel (asterisks) including 3D reconstruction of the bifurcation site **(F')**.

In the study by Feng et al., authors studied the prevalence of healed plaques and their characteristics by analyzing the findings of 13 studies using optical coherence tomography (OCT) through a formal systematic review, meta-analysis and meta-regression. The main finding was that the incidence of healed plaques identified by OCT was higher in patients with stable angina pectoris than in patients with acute coronary syndrome (ACS), and was higher in culprit plaques than in non-culprit. Interestingly, in the healed plaque group, higher incidences of thin-cap fibro-atheroma (TCFA), plaque rupture, micro-vessels, macrophage accumulation, and calcification were found.

Kei-Yan Ng et al. proposed the hypothesis that intracoronary imaging may improve outcomes at an individual level, but paradoxically worsen outcomes for patients receiving angiography-guided PCI in centers with a high proportion of imaging use. This controversial hypothesis

originated from the discrepancy seen between observational studies and randomized trials. In the authors' opinion, such differences might be explained by the fact that operators become over reliant on imaging guidance and less familiar with angiography-guided PCI. Moreover, they found that higher background imaging rate was associated with a paradoxical increase in all-cause and cardiovascular mortality rates at 1 year in patients undergoing angiography- guided PCI and refer to this phenomenon as the “imaging paradox.”

Amato et al. designed a proof-of-concept study assessing the potential of active connection matrixes (ACMs) to increase measurement precision of morphological structures (e.g., stenosis and lumen diameter) and to grasp morphological features (arterial walls) from quantitative coronary angiography (QCA), not detectable on the original images. ACMs are unsupervised artificial adaptive systems able to extract features of interest (edges, tissue differentiation, etc.) from digital images unnoticeable with conventional systems. The results demonstrated that ACMs increase the measurement precision of coronary lumen diameter allowing extraction of hidden features from QCA images that mirror well the arterial wall derived by IVUS.

The study by Lavarra et al. proposed a technical refinement, the Proximal Side Optimization Technique (PSO), aiming at improving the procedural strategy during double-kissing crush (DK crush) stenting. This step consists in post-dilating the drug-eluting stent positioned in the side branch (SB DES) with the delivery balloon, pulled back halfway in the main branch followed by further post-dilation with a 0.5 mm larger non-compliant balloon prior to the crush step. OCT 3D reconstruction demonstrated an adequate SB DES expansion and apposition before it being crushed, with larger Space of Optimal Wiring (SOW) when compared to the non-PSO treated group in terms of area, eliminating the residual space between stent and vessel wall and the risk for inadequate wire passage during rewiring.

Barbieri et al. offered an overview on the role of OCT in the evaluation of Spontaneous Coronary Artery Dissections (SCAD). Authors highlighted the importance of balancing the diagnostic benefits of this tool vs. the procedural risks. Among the strengths of OCT, they underlined the superior spatial resolution when compared to intravascular ultrasound (IVUS) with an evident advantage in the diagnosis of SCAD for the identification of intramural hematoma, endothelial tears, or entry sites of dissections. On the other hand, the main drawback related to OCT is the possibility of progression of false lumen due to the contrast injection required for imaging acquisition, especially in tortuous vessels. However, OCT is considered an important resource during the diagnostic process of SCAD, allowing the finetuning of the procedural strategy when a PCI is considered.

Hu et al. performed a network meta-analysis of 28 randomized trials and 11,860 patients undergoing PCI assisted by different modalities (IVUS, OCT, FFR/QFR) in the DES era. Authors aimed to evaluate the impact of such different PCI guidance modalities on clinical outcomes. According to this analysis, IVUS led to lower risks of MACE than coronary angiography, mainly due to lower risks of cardiovascular death and TVR/TLR, showing a trend toward decreased risk of stent thrombosis. Another interesting finding was that hemodynamic parameter (FFR/QFR)-guided PCI could significantly reduce stroke risk compared with angiography, IVUS and OCT.

Alasnag et al. provided a compelling review on the intracoronary imaging-guidance during PCI procedures. Authors analyze the causes of the poor penetration of such techniques into the clinical practice worldwide, offering a wide spectrum of possible explanations with a special mention to the lack of education and the associated challenges of interpretation. Interestingly, authors also focus on plaque characterization with a high-quality gallery of images that we believe will be useful to the readers.

In their prospective, multicenter, randomized, non-inferiority study, Tao et al. compared a biodegradable polymer sirolimus-eluting stent to a durable polymer everolimus-eluting stent in the ST- Elevation myocardial infarction (STEMI) setting. In particular, authors evaluated the vascular healing of the two platforms in terms of neointimal thickness (NIT) at 6 months with OCT. Authors found the biodegradable polymer sirolimus-eluting stent arm to be non-inferior to the durable polymer everolimus-eluting stent arm with a percentage of uncovered struts of 0.55% vs. 0.40%. Also, at 1 year of follow-up, no significant differences in the incidence of target lesion failure or definite, probable, or possible ST events were found between groups.

In conclusion, ICI offers impressive pathophysiological insights that were not possible to obtain *in vivo* before its introduction in the clinical scenario. Moreover, the procedural and clinical usefulness of ICI has been widely demonstrated to date and several appropriately-sized trials are paving the way for its more extensive use that, together with the tools for the functional assessment of the stenosis severity, is expected to further improve the outcomes of patients undergoing PCIs.

## Author Contributions

IK, PC, and AM critically revised the manuscript draft and constructed the figure. All authors contributed to the article and approved the submitted version.

## Conflict of Interest

The authors declare that the research was conducted in the absence of any commercial or financial relationships that could be construed as a potential conflict of interest.

## Publisher's Note

All claims expressed in this article are solely those of the authors and do not necessarily represent those of their affiliated organizations, or those of the publisher, the editors and the reviewers. Any product that may be evaluated in this article, or claim that may be made by its manufacturer, is not guaranteed or endorsed by the publisher.

